# Clinical evaluation of visual snow syndrome: a structured narrative review with proposed clinical diagnostic approach

**DOI:** 10.3389/fneur.2026.1961508

**Published:** 2026-10-12

**Authors:** Nemira Žilinskaitė-Tranas, Gytis Makarevičius, Aušra Liveikienė, Kristina Ryliškienė

**Affiliations:** 1 Vilnius University, Faculty of Medicine, Vilnius, Lithuania; 2 Vilnius University, Medical Faculty, Institute of Clinical Medicine, Neurology and Neurosurgery Clinic, Vilnius, Lithuania; 3 Hila Clinic, Vilnius, Lithuania

**Keywords:** cortical hyperexcitability, diagnostic approach, differential diagnosis, functional neuroimaging, neuro-ophthalmology, thalamocortical dysrhythmia, visual snow syndrome

## Abstract

Visual snow syndrome (VSS) is a chronic neurological condition marked by persistent, bilateral, pan-field visual static and a constellation of additional visual and non-visual symptoms. Affecting an estimate of 2% of the populations, VSS remains substantially underdiagnosed, as its symptoms overlap with migraine aura and structural ophthalmological disease, and no standardised diagnostic protocol currently exists. This structured narrative review synthesises evidence on the clinical features, pathophysiology, comorbidities, and differential diagnosis. It also proposes a stepwise diagnostic approach for routine practice, distinguished from the advanced, research-oriented evaluations used to investigate underlying mechanisms. Converging neuroimaging and electrophysiological evidence suggests cortical hyperexcitability and large-scale network dysfunction may underlie VSS, though the precise mechanisms remain incompletely understood. We recommend a multidisciplinary, patient-centred management approach and identify biomarker development, multicentre validation of the diagnostic approach, and large-scale interventional trials as research priorities.

## 1 Introduction

Visual snow syndrome (VSS) is a chronic neurological condition characterised by the continuous perception of bilateral, pan-field visual static. Patients widely relate it to the noise of an analog television screen (1). The visual ‘snow’ consists of tiny flickering dots that vary in colour, density, and perceived intensity. They persist regardless of ambient illumination and are present with eyes closed (2). This non-episodic, continuous character distinguishes VSS from episodic visual phenomena such as migraine aura, occipital seizures, and transient visual obscurations (3, 4).

The functional consequences of VSS are substantial. Persistent visual disturbance impairs reading, screen use, navigation in low-contrast environments, driving, and social participation (5). Difficulties are amplified in environments with uniform or plain backgrounds, therefore symptoms generate cumulative psychological burden (6). From an epidemiological perspective, VSS has been estimated to affect approximately 2% of the general population. This places VSS among the more prevalent neurological conditions encountered in clinical practice, yet it remains largely unrecognised (7, 8).

Recognition of VSS is essential for appropriate patient care. Misdiagnosis may lead to unnecessary investigations, reinforce diagnostic uncertainty, and generate treatment-induced anxiety (6, 9). Diagnosis currently relies on clinical pattern recognition, symptom characterisation, and systematic exclusion of secondary aetiologies. No pathognomonic biomarkers and consistent structural neuroimaging abnormalities exist to confirm the diagnosis directly (10). VSS may additionally represent a broader disorder of multisensory network regulation. Conceptualisation has implications for both patient management and for understanding central sensory processing disorders more broadly (11).

Despite growing scientific interest, no universally accepted standardised diagnostic protocol exists for VSS (2). Without structured guidance, clinicians may apply redundant investigations, or reinforce patient distress through diagnostic delay. This structured narrative review addresses that gap by providing a clinically focused synthesis of VSS’s definition, pathophysiology, symptoms, comorbidities, secondary causes, and diagnostic testing. It also presents a structured diagnostic approach that distinguishes routine clinical investigations from advanced, research-oriented ones.

## 2 Materials and methods

### 2.1 Study design

This study was conducted as a narrative review employing a systematic search strategy and qualitative thematic synthesis, rather than a formal systematic review. A narrative synthesis approach was selected because the VSS literature is characterised by considerable methodological heterogeneity and a predominance of small observational studies, case series, and single-centre imaging studies. Formal meta-analysis was therefore not feasible (12, 13). The review was designed to provide a clinically actionable synthesis of current evidence, rather than a statistical pooling of results.

### 2.2 Database and search strategy

A structured literature search was conducted across three databases: PubMed/MEDLINE, Scopus, and Web of Science, using date- and language-restricted, field-appropriate translations of a single search string (see below), covering the period January 2020 to 10 April 2026, in English.

The search was restricted to January 2020 onward to focus on literature published following the establishment of formal ICHD-3 diagnostic criteria for visual snow syndrome (2018). This allowed the review to prioritise studies applying a consistent, standardised case definition. Foundational pre-2020 literature establishing this diagnostic framework, such as including the original description distinguishing VSS from persistent migraine aura (14), the proposed diagnostic criteria (15), and early electrophysiological findings (16), was incorporated through manual reference tracking.

In PubMed/MEDLINE, a total of 192 records were identified with no date or filter restrictions applied. Following application of the date restriction, 143 records were retained. The full search string, incorporating publication type filters, English language restriction, and all MeSH/keyword terms, was then applied, yielding 29 records. All 29 records were retrieved in full text and included in the review. None were excluded at full-text stage. Three additional records predating 2020 were identified through manual reference tracking. Together with already selected pre-2020 literature, this yielded 32 primary sources forming the core evidence base for the review.

To verify the comprehensiveness of this core search, the same date- and language-restricted search was additionally run in Scopus and Web of Science, and re-run in PubMed/MEDLINE. It was run without the publication-type restriction, to allow direct comparison across databases on equal terms. Results are current as of 2 September 2026. Embase was not searched directly, as our institution does not hold a direct subscription to the platform. The selection process is summarised in Table 1. The search string for each database is specified in the Supplementary document.

**Table 1:** Literature selection flow.

Stage	Action	Records
Identification	PubMed/MEDLINE, no filters, all dates	192
Date restriction	Limited to Jan 2020–10 Apr 2026	143
Structured search (core evidence base)	Full search string + publication-type + language filters (PubMed only)	29
Manual additions	Foundational pre-2020 references via reference tracking	+3
Core primary sources		32
Type-filter gap review	Imaging/electrophysiology records within the unfiltered 128-record PubMed search excluded by the publication-type filter, re-screened	21
Already covered by existing citations		9
Core sources recovered	Primary neuroimaging/electrophysiology studies added	12
Cross-database verification	PubMed (no type filter) 128 + Scopus 161 + Web of Science 123	412
Deduplication + date-cutoff check	Cross-database duplicates removed; post-10-Apr-2026 records excluded	176
Already represented in PubMed search	Overlap with the 128-record PubMed set	128
New records screened	Records unique to Scopus/Web of Science	48
Screening outcome	Records met topical/content-type eligibility but were judged, on full-text review, not to alter the scope or conclusions of the review	20
Total primary sources informing the review	Core (32) + imaging recovery (12)	44

### 2.3 Selection process

Records identified by the search were exported and processed in sequential stages. Duplicate records were removed prior to screening. Titles and abstracts of all remaining records were independently screened by two reviewers (NZ-T and GM) for relevance against the predefined eligibility criteria across five domains: clinical presentation and diagnostic criteria of VSS, pathophysiological mechanisms, including neuroimaging and electrophysiological data, comorbidities and associated conditions, differential diagnosis and secondary causes, and instrumental diagnostic modalities. Disagreements between reviewers were resolved by a third author (KR). Records meeting eligibility criteria at title/abstract screening underwent full-text review. Studies were excluded if they were not retrievable in full text, addressed pharmacological intervention without diagnostic relevance, or were superseded by a more comprehensive publication from the same research group. Existing reviews and syntheses of the VSS literature were not excluded from consideration. Where relevant, they were retained and cited as contextual or comparative sources rather than treated as primary evidence.

### 2.4 Data synthesis

Evidence was synthesised using a qualitative thematic approach, organised into predefined analytical domains: definition and pathophysiology, clinical presentation, comorbidities, secondary causes and differential diagnosis and instrumental evaluation. Emphasis was placed on distinguishing investigations applicable in routine clinical practice from those appropriate only to research or complex diagnostic contexts. The structured diagnostic approach presented herein represents an evidence-informed, author-derived integration of current data, rather than a formal Delphi-process consensus guideline. It is intended as a pragmatic clinical tool pending multicentre prospective validation.

Given the narrative synthesis design of this review, formal per-study risk-of-bias scoring using tools such as AXIS was not undertaken, as the heterogeneity of included study designs (cross-sectional surveys, case–control imaging studies, case series, and existing systematic/narrative reviews) does not lend itself to a single standardised checklist. Instead, methodological rigour and sample size were considered qualitatively during synthesis, and reporting quality of the review itself was assessed using the Scale for the Assessment of Narrative Review Articles (SANRA), the results of which are presented in Supplementary Tables 2.

## 3 Literature review

### 3.1 Definition and pathophysiology

Visual snow syndrome is defined by the International Classification of Headache Disorders, Third Edition (ICHD-3), as a condition characterised by continuous, dynamic, flickering tiny dots across the entire visual field persisting for a minimum of 3 months. This definition explicitly excludes cases attributable to structural pathology of the visual pathways or any other neurological disorder that could better account for the symptoms (17). Diagnosis additionally requires at least two of the following associated phenomena: palinopsia, enhanced entoptic phenomena, photophobia, or impaired night vision (15). Critically, symptoms must not be attributable solely to migraine aura, ophthalmological disease or HPPD (1, 12) (Figure 1).

**Figure 1:**
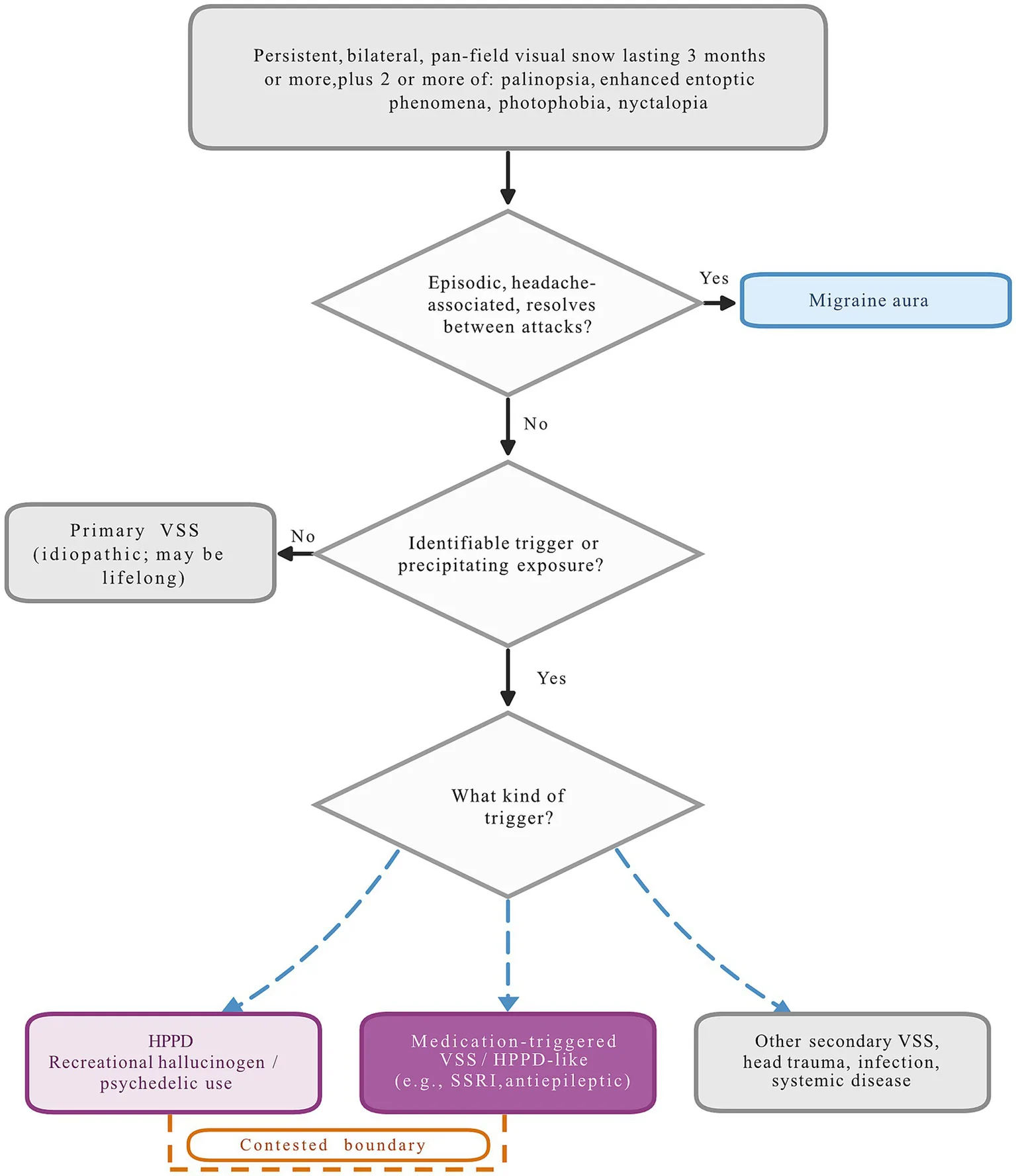
Trigger-based classification pathway for visual snow syndrome (VSS), hallucinogen persisting perception disorder (HPPD), and migraine aura. Classification begins with the core ICHD-3 symptom criteria, first distinguishing episodic migraine aura from persistent visual snow, then separating primary (idiopathic) VSS from secondary presentations by the presence and type of an identifiable trigger. The boundary between recreational hallucinogen-triggered HPPD and prescription medication-triggered VSS/HPPD-like presentations is contested, reflecting overlapping receptor mechanisms and potential non-disclosure of recreational substance use or delayed recognition of lifelong symptoms (2, 10, 39, 40, 52). VSS, Visual Snow Syndrome; HPPD, Hallucinogen Persisting Perception Disorder; SSRI, selective serotonin reuptake inhibitor (4, 25).

The pathophysiology of VSS remains incompletely understood (2, 18). Converging neuroimaging and electrophysiological evidence has led to hypotheses centred on cortical hyperexcitability and thalamocortical network dysfunction, although these proposals require further mechanistic investigation before they can be considered established (19). A single-centre 18F-fluorodeoxyglucose positron emission tomography (18F-FDG PET) study reported focal hypermetabolism in the right lingual gyrus and anterior cerebellum at rest in VSS patients relative to healthy controls (20). This finding proposed to reflect localised cortical hyperexcitability, but independent replication is still required (Figure 2). Visual evoked potential (VEP) studies have reported impaired habituation of the P1 component in some VSS patients (16, 21). This has been interpreted as possibly reflecting disrupted excitation-inhibition (E/I) balance in the visual cortex, though findings have not been consistent across studies.

**Figure 2:**
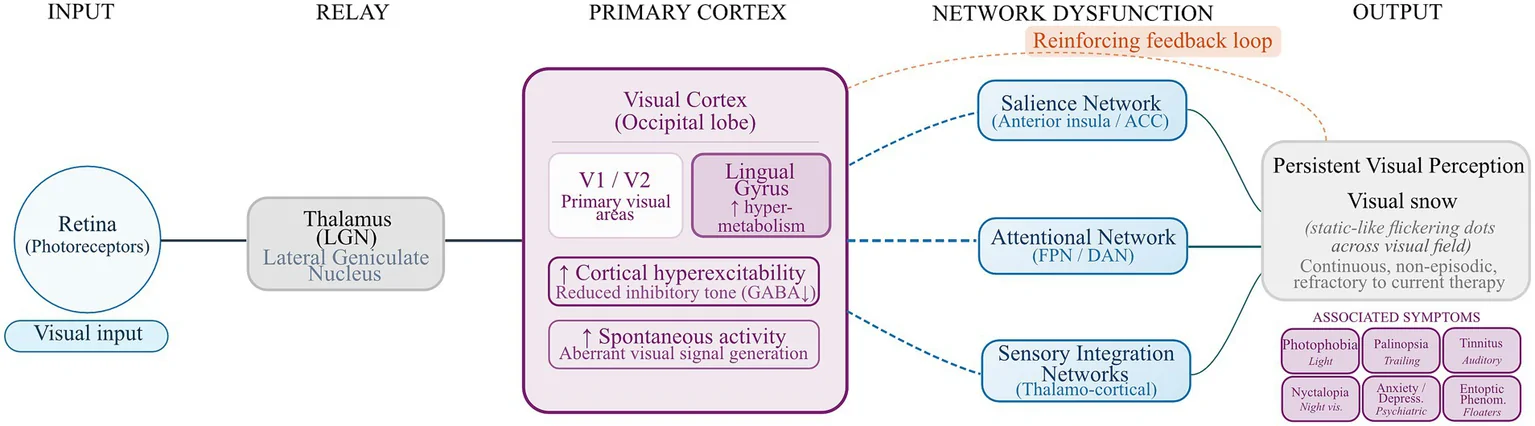
Plausible pathophysiological hypothesis of visual snow syndrome (VSS). ACC, anterior cingulate cortex; DAN, dorsal attention network; FPN, frontoparietal network; GABA, gamma-aminobutyric acid; LGN, lateral geniculate nucleus; PET, positron emission tomography; VSS, Visual Snow Syndrome (11, 13, 16, 18, 20, 21, 26, 53).

Resting-state functional MRI has been reported to demonstrate altered functional connectivity in VSS, with abnormalities identified within the visual, attentional, salience, and default mode networks in several studies (22–24). These findings have led to the proposal of VSS as a network disorder rather than a circumscribed cortical lesion condition (11, 25), although this conceptualisation remains based on limited evidence. Magnetoencephalography (MEG) has identified cortical oscillatory dysrhythmia in VSS (26). Anomalous power distributions in alpha and gamma frequency bands have been proposed to be consistent with thalamocortical dysrhythmia, a model originally advanced to explain chronic positive sensory phenomena in pain and tinnitus (11, 25). Glutamatergic and serotonergic dysregulation have been proposed as contributors to the excitation/inhibition imbalance in VSS (18, 27), and microstructural white matter alterations in visual and attentional pathways have been reported in some imaging studies (28–30), but the significance of these findings remains to be established in larger cohorts. Collectively, available evidence is consistent with, but does not yet firmly establish, a model of VSS as a disorder of central visual processing characterised by cortical hyperexcitability and large-scale network dysconnectivity without primary retinal or optic nerve pathology (1, 19).

### 3.2 Clinical presentation

#### 3.2.1 Core visual symptoms

The defining feature of VSS is persistent, continuous, bilateral, pan-field visual static consisting of tiny flickering dots present in all lighting conditions and with eyes closed (1). Dots vary in colour, density, and perceived intensity. Beyond visual snow itself, the syndrome is characterised by a reproducible constellation of additional visual phenomena summarised in Table 2.

**Table 2:** Core visual and non-visual symptoms of visual snow syndrome: clinical description and functional impact.

Symptom	Clinical description	Functional impact
Visual symptoms		
Visual snow	Continuous bilateral pan-field flickering dots in all lighting conditions and with eyes closed; variable colour and density	Universal impairment of visual clarity; cumulative sensory fatigue (1)
Palinopsia	Prolonged afterimages after stimulus removal; visual trailing of moving objects	Visual clutter; impaired reading in bright environments; difficulty with dynamic scenes (31)
Enhanced Entoptic Phenomena	Exaggerated perception of floaters and blue field entoptic phenomenon (Scheerer’s phenomenon)	Persistent visual distraction; awareness of normally suppressed percepts (12)
Photopsias	Spontaneous phosphene-like flashes without external stimulus; variable frequency	Sensory discomfort, particularly in dark environments (32)
Photophobia	Pathological light sensitivity causing discomfort or pain under normal illumination; persistent and non-episodic in character	Limits outdoor activity, screen use, and social participation (13, 33)
Nyctalopia	Impaired visual function under scotopic conditions; not attributable to retinal photoreceptor pathology in primary VSS	Restricts nocturnal navigation and driving (33)
Non-visual symptoms		
Tinnitus	Subjective auditory phantom perception (ringing, buzzing, or hissing) without an external sound source; frequently bilateral and persistent, paralleling the continuous character of visual snow	Reported in up to 75% of VSS cohorts; proposed to share a mechanism of multisensory cortical hyperexcitability with visual symptoms (12)
Depersonalisation/derealisation	Altered subjective experience of self (depersonalisation) or of one’s surroundings (derealisation), often described as feeling detached or observing oneself from outside	Among the most commonly reported and most distressing associated symptoms; contributes substantially to psychological burden (5)
Fatigue	Physical and cognitive fatigue, including concentration impairment and slowed processing, plausibly related to the sustained attentional demands of processing aberrant visual input	Reduces occupational and leisure functioning; contributes to deterioration in psychological wellbeing (5)
Anxiety/depression	Psychiatric comorbidity of variable severity; directionality of the association with VSS symptoms is unclear and may be bidirectional	Reported in up to 72% (anxiety) and 52–79% (depression) of VSS patients; multidisciplinary mental health assessment is recommended (5)

VSS, Visual Snow Syndrome.

Palinopsia, encompassing prolonged afterimages and visual trailing, is among the most prevalent associated features (31). Heightened awareness of entoptic phenomena is also characteristic, including exaggerated perception of floaters and the blue field entoptic phenomenon (Scheerer’s phenomenon) (12). Photopsia have been reported with variable frequency (32).

Nyctalopia significantly exacerbates functional impact by restricting nocturnal navigation (33). Photophobia has been identified as one of the most disabling symptoms in VSS (19, 33). The cumulative functional impact is substantial, with significant impairment in occupational and leisure functioning reported by many patients (5). However, these functional impacts are not universally experienced as negative: some individuals report minimal distress or even a degree of fascination with their visual symptoms, particularly among those with long-standing, lifelong presentations (34).

#### 3.2.2 Additional non-visual symptoms

VSS involves significant non-visual symptomatology reflecting a broader disturbance of sensory processing. Tinnitus has been reported in up to 75% of VSS patients in some series (12). The co-occurrence of auditory and visual phantom phenomena has been proposed to share a mechanism of multisensory cortical hyperexcitability (11, 25), a hypothesis supported by MEG findings of cortical oscillatory dysrhythmia (26), but the causal relationship between these findings remains uncertain. Fatigue and cognitive symptoms, including concentration impairment and slowed processing, are reported by many patients and may be related to the sustained attentional demands of processing aberrant visual input. Dizziness and headache, independent of migraine, may accompany VSS and may contribute to its clinical overlap with other perceptual disorders (11, 25). Depersonalisation and derealisation are also reported in VSS and are frequently rated among the most distressing associated symptoms, independent of visual symptom severity (Table 2). Clinicians should ask about them explicitly, as patients often do not volunteer them unprompted.

### 3.3 Comorbidities

#### 3.3.1 Migraine

Migraine is the most frequently reported comorbidity in VSS, occurring in 57–68% of patients across large cohort studies (1, 14). The relationship between migraine and VSS is complex. Migraine-specific therapies, including triptans and preventive agents, have not been shown to consistently ameliorate VSS symptoms (35, 36), which has been interpreted as suggesting at least partially divergent pathophysiological mechanisms. Photophobia is a feature shared by both conditions (13), and shared disruption of retino-thalamo-cortical pathways has been proposed as a potential explanation (3), though this remains speculative. VSS-associated photophobia is distinguished from migraines’ photophobia by its persistent, non-episodic character. A multicentre observational study found that 7.8% of patients attending headache units with migraine fulfilled full VSS criteria, with an additional 26.7% demonstrating episodic visual snow features (37), suggesting the clinical spectrum of VSS may be broader than previously recognised. Available neuroimaging and electrophysiological evidence has been interpreted as supporting VSS as a distinct entity from migraine with aura, characterised by persistent network-level abnormalities rather than the propagating cortical spreading depression of migraine aura (3, 4).

#### 3.3.2 Anxiety and depression

Anxiety has been reported in up to 72% of VSS patients, and depression in 52–79%, in clinical cohort studies (5, 38). The directionality of the association between VSS and these psychiatric symptoms is unclear. Shared mechanisms are likely and both bidirectional and independent relationships have been proposed (5). The chronic, intrusive, and treatment-refractory nature of VSS symptoms may contribute to psychological distress. Conversely, pre-existing anxiety and depression may amplify symptom perception through attentional sensitisation mechanisms. This interaction is consistent with models of central sensitisation in which affective dysregulation lowers sensory filtering thresholds, though whether this mechanism is causally operative in VSS has not been established. Assessment of mental health is warranted in all VSS patients given the frequency of psychiatric comorbidity, and multidisciplinary management incorporating psychiatry or clinical psychology is recommended.

#### 3.3.3 Tinnitus, persistent postural-perceptual dizziness and fibromyalgia

Chronic tinnitus co-occurs with VSS at rates substantially exceeding general population estimates, with reported prevalence ranging from 54 to 75% in VSS cohorts (12). The co-occurrence of VSS with tinnitus, persistent postural-perceptual dizziness and fibromyalgia has been proposed to reflect shared dysfunction of sensory processing networks across multiple modalities (11, 25). Each of these conditions has been associated with abnormalities in sensory and attentional network function, and the hypothesis of a shared thalamocortical dysrhythmia mechanism has been advanced, although direct mechanistic evidence in VSS specifically remains limited. Migraine has been proposed as a common predisposing risk factor across this putative spectrum of perceptual disorders (11, 25).

#### 3.3.4 Fatigue

Significant physical and cognitive fatigue is reported by a substantial proportion of VSS patients (5). Cognitive fatigue may in part reflect the sustained attentional resources required to process and partially suppress aberrant visual input, but this hypothesis has not been directly tested. Fatigue contributes to reduced occupational functioning and deterioration in psychological wellbeing, reinforcing the multidimensional nature of VSS-associated functional impact.

### 3.4 Secondary causes and differential diagnosis

VSS is a diagnosis of exclusion, and evaluation for secondary aetiologies should be pursued systematically in patients presenting with visual snow-like symptoms for the first time, or with atypical, evolving, or red-flag features (6, 9). In patients with a long-standing, stable presentation meeting the ICHD-3 criteria and no such features, the yield of repeat or exhaustive secondary work-up is low, and evaluation can be tailored accordingly. Differential diagnosis of VSS is summarised in Table 3.

**Table 3:** Differential diagnosis of visual snow syndrome: symptom pattern, temporal profile, red flags, and key exclusion investigations (4, 6, 9, 15, 32, 35, 40).

Condition	Prevalence	Symptom pattern	Temporal Profile	Red flags	Exam/Imaging findings	Key exclusion tests
Primary VSS						
Primary VSS	~2–3% (7, 8)	Bilateral pan-field static; palinopsia; photophobia; entoptic phenomena	Persistent, ≥3 months, non-progressive	None if presentation is typical	Normal structural MRI; normal fundoscopy and OCT	Clinical diagnosis; MRI to exclude secondary causes
Neurological						
Migraine with Aura	10–15% (52)	Positive visual phenomena (fortification spectra, scintillations, scotomata)	Episodic, minutes–hours; resolves between attacks	New-onset aura without prior headache; aura duration >1 h	Normal between attacks; MRI may show WM changes	Clinical history; MRI if atypical features present
Persistent Migraine Aura	Rare	Continuous aura-like phenomena following a migraine episode	Persistent after index migraine; may last weeks to months	Change in aura character; neurological deficits	Usually normal MRI; exclude structural cause	MRI; exclude ischaemic stroke; EEG
Occipital/ Posterior Cortical Lesion	NA	Visual field defects; complex visual hallucinations; metamorphopsia	Acute or subacute onset; may be progressive	Homonymous hemianopia; neurological signs; acute onset	MRI: focal posterior cortex or optic radiation abnormality	MRI brain with gadolinium; CSF if inflammatory aetiology suspected
Visual Seizures (Occipital Epilepsy)	5–10% of all epileptic cases (54)	Brief stereotyped coloured circles or shapes; ictal blindness	Seconds to minutes; recurrent	Loss of awareness; automatisms; post-ictal confusion	EEG: occipital epileptiform discharges; MRI often normal	EEG (including sleep-deprived or prolonged recording); MRI brain
Idiopathic Intracranial Hypertension	~1–3/100,000 general population (55)	Visual obscurations; transient visual loss; pulsatile tinnitus; headache	Subacute; progressive if untreated	Papilloedema; progressive visual field loss; headache worse on Valsalva	MRI: empty sella, optic nerve sheath dilation, posterior scleral flattening; papilloedema on fundoscopy	MRI brain and orbits; lumbar puncture (opening pressure); fundoscopy with visual fields
Ophtamological						
Optic Neuropathy (e.g., LHON)	~1/27,000–1/45,000 (56)	Visual loss; decreased acuity; colour vision deficit; central scotoma	Subacute or progressive; may stabilise	Painful eye movement; RAPD; progressive visual loss	Optic disc pallor; RNFL thinning on OCT; possible MRI optic nerve signal change	OCT; VEP (delayed P100); MRI orbits and brain; genetic testing for LHON
Retinal Disease (e.g., RP, macular dystrophy)	~1/4,000 (57)	Photopsias, floaters, visual distortion; peripheral field loss in RP	Variable; often progressive	Sudden visual loss; progressive field constriction; family history	Abnormal fundoscopy; OCT structural changes	OCT; ERG; fundus autofluorescence; genetic panel
Substance-related						
HPPD (recreational)	~4.2% of hallucinogen users (58)	Visual trails, afterimages, geometric hallucinations, visual snow	Persistent after hallucinogen use; may be delayed in onset	History of hallucinogen or psychedelic substance exposure	Normal examination; normal MRI	Careful, non-judgemental substance-use history; urine toxicology where recent use is suspected; psychiatric assessment
Medication-triggered VSS/HPPD-like	Not systematically established	Clinically indistinguishable from primary VSS or recreational HPPD	Onset temporally associated with initiation, dose change, or discontinuation of an implicated agent.	Temporal relationship to a new or changed medication; no recreational substance use disclosed	Normal examination; normal MRI	Detailed, dated medication history including recent changes; consider trial of dose adjustment/discontinuation where clinically appropriate; toxicology screen to help exclude concurrent recreational use

VSS, Visual Snow Syndrome; HPPD, Hallucinogen Persisting Perception Disorder; LHON, Leber Hereditary Optic Neuropathy; RP, Retinitis Pigmentosa; RAPD, Relative Afferent Pupillary Defect; OCT, Optical Coherence Tomography; RNFL, Retinal Nerve Fibre Layer; ERG, Electroretinography; VEP, Visual Evoked Potentials; MRI, Magnetic Resonance Imaging; EEG, Electroencephalography; WM, White Matter; CSF, Cerebrospinal Fluid; BOLD, Blood-Oxygen-Level-Dependent.

HPPD needs a clinical differential diagnosis (39). HPPD occurs in the context of prior hallucinogen exposure and may present with visual snow, palinopsia, and geometric visual disturbances. It is more prevalent in males typically presents at a later age than primary VSS, and may include visual flashback phenomena not characteristic of primary VSS (39). Persistent visual disturbances following use of prescription medications, including antidepressants (trazodone, nefazodone, mirtazapine) and antiepileptic agents (topiramate), have also been reported and should be considered (2, 10, 40). The boundary between these categories is not always clear-cut in practice: patients may be reluctant to disclose recreational hallucinogen use, and individuals with primary VSS may come to clinical attention only after starting a medication. This produced an apparent but not necessarily causal temporal association. Because symptom phenomenology overlaps substantially across HPPD, medication-triggered presentations, and primary VSS, a detailed and non-judgemental history covering both recreational substance use and medication timing is often more informative than examination or imaging, which are typically normal in all three.

Optic neuropathies, including Leber Hereditary Optic Neuropathy and toxic optic neuropathy, are distinguished from primary VSS by the presence of reduced visual acuity, colour vision deficit, optic disc changes and optic nerve attenuation on OCT. (41, 42) Retinal diseases are identified by characteristic findings on fundoscopy, ERG, and OCT. Occipital lobe lesions and cerebral venous thrombosis may be excluded by MRI brain with dedicated sequences, while idiopathic intracranial hypertension (IIH) requires clinical assessment, fundoscopic evaluation (e.g., papilledema), and confirmation by elevated opening pressure on lumbar puncture. Visual seizures are distinguishable from VSS by their episodic, stereotyped character and EEG abnormalities (Table 4).

**Table 4:** Distinguishing features of primary versus secondary visual snow syndrome (11, 20, 44).

Feature	Primary VSS	Secondary VSS
Symptom onset	Gradual; typically, in adolescence or early adulthood; may follow a first migraine episode or psychosocial stressor; can be lifelong (34)	Sudden or temporally linked to a precipitant: hallucinogen use, head trauma, medication initiation, or systemic illness
Temporal course	Chronic and continuous (≥3 months); non-progressive in the majority of cases	Variable; may be progressive, episodic, or fluctuating depending on the underlying aetiology
Associated symptoms	Palinopsia, enhanced entoptic phenomena, photophobia, nyctalopia, tinnitus, fatigue; anxiety and depression frequently reported	Aetiology-dependent: e.g., visual loss in optic neuropathy; peripheral field defects in retinal disease; raised intracranial pressure features in IIH
Structural neuroimaging	Normal on conventional MRI; research-level functional imaging has reported lingual gyrus hypermetabolism and altered network connectivity in some studies	May show focal lesions, optic nerve changes, posterior cortex abnormality, or features of IIH
Ophthalmological findings	Normal OCT, fundoscopy; ERG and VEP typically within normal limits in primary VSS, though subtle changes have been reported in some studies	Potentially abnormal optic disc pallor, retinal structural atrophy, ERG amplitude reduction, VEP latency delay
Identifiable precipitating exposure	Absent; no identifiable toxic, traumatic, or infectious trigger	Present: hallucinogen or recreational drug use (HPPD), medication (antidepressants, antiepileptics), head trauma, infection, or systemic disease
Response to treatment	Limited response to pharmacotherapy; no disease-modifying treatment currently established	May improve with treatment of the underlying condition (e.g., cessation of offending agent; management of raised intracranial pressure)

VSS, Visual Snow Syndrome; HPPD, Hallucinogen Persisting Perception Disorder; OCT, Optical Coherence Tomography; ERG, Electroretinography; VEP, Visual Evoked Potentials; IIH, Idiopathic Intracranial Hypertension; fMRI, Functional Magnetic Resonance Imaging; PET, Positron Emission Tomography.

Red flags warranting urgent neuroimaging and specialist review include new-onset or intermittent visual snow, unilateral or quadrantanopic distribution, associated acute neurological deficits, painful eye movement and sudden-onset symptoms (9).

### 3.5 Structured diagnostic approach

Establishing the diagnosis of VSS requires a structured, stepwise approach that systematically excludes secondary aetiologies before a primary diagnosis can be made. Investigations applicable in routine clinical practice are distinguished here from those reserved for advanced, research-oriented evaluation. Advanced neuroimaging and electrophysiological investigations (including fMRI, 18F-FDG PET, and MEG) are not part of the routine diagnostic workup for VSS. Their role is to characterise pathophysiological mechanisms in primary VSS within research contexts, or to assist diagnostic classification in selected complex cases.

#### 3.5.1 Routine clinical diagnostic evaluation

The following four-step pathway is not intended to be applied uniformly to every patient presenting with VSS. Kondziella et al. note that patients with long-standing, stable, and clearly recognisable VSS often seek clinical reassurance rather than further diagnostic evaluation, and that extensive investigation in this group carries a risk of provoking anxiety without a corresponding diagnostic yield (43). Accordingly, for patients with a stable presentation meeting the criteria in full, no red flags (Table 4), and no atypical features, Step 1 alone may be sufficient, with further steps offered selectively rather than performed routinely. The full pathway (Steps 1–4) is indicated for new-onset presentations, atypical or evolving symptoms, or where red flags or diagnostic uncertainty are present.

Step 1: Clinical History and Neurological Examination. A detailed clinical history is the critical foundation of VSS assessment. Clinicians should systematically characterise: (1) the nature, onset, duration, and progression of visual symptoms, with particular attention to the bilateral, pan-field, continuous quality of visual snow and the presence of associated visual phenomena (palinopsia, photophobia, entoptic phenomena, nyctalopia); (2) prior exposure to hallucinogens, psychedelic substances, or medications that may precipitate HPPD or drug-induced visual disturbances (2); (3) history of head trauma, meningitis, or systemic disease; (4) family history of neurological or ophthalmological conditions; and (5) psychiatric comorbidity, including anxiety, depression, and depersonalisation (5). The symptom duration criterion of ≥3 months must be explicitly established (17) (Table 5). Red flags (unilateral distribution, acute onset, progressive course, painful eye movement, associated neurological deficits) mandate expedited assessment and should lower the threshold for urgent brain MRI (6, 9). Neurological examination including assessment of visual acuity, colour vision, visual fields by confrontation, pupillary responses (with attention to relative afferent pupillary defect), and fundoscopy is mandatory.

**Table 5:** Diagnostic criteria for visual snow syndrome (ICHD-3 Appendix) with clinical commentary (15, 17, 35).

Criterion	Specification	Clinical commentary
A. Persistent visual snow	Continuous, dynamic, tiny flickering dots across the entire visual field for ≥3 months; present in all lighting conditions including with eyes closed; variable colour and density	Must be bilateral and pan-field; unilateral or quadrantanopic symptoms require urgent neuroimaging to exclude structural pathology
B. ≥2 Additional visual symptoms	At least two of: (1) palinopsia; (2) enhanced entoptic phenomena (floaters, blue field entoptic phenomenon, photopsias); (3) photophobia; (4) nyctalopia	Symptoms must be present independently of migraine attacks; visual phenomena isolated to the migraine cycle do not fulfil criterion B for primary VSS
C. Exclusion of other causes	Symptoms are not better accounted for by: migraine aura alone; ophthalmological pathology (optic neuropathy, retinal disease); HPPD; medication effect; or any other neurological or systemic disorder	Requires structured diagnostic evaluation. Absence of red flags and normal ophthalmological and structural neuroimaging assessment support a primary diagnosis
D. Non-progressive course*	Symptoms do not demonstrate significant worsening over time; stability distinguishes VSS from progressive retinal or neurological pathology	Acute deterioration or emergence of new neurological signs at any stage of follow-up warrants re-evaluation for a secondary cause

*Criterion D represents an author-proposed clinical supplement to the ICHD-3 diagnostic framework. Source: ICHD-3 Appendix (1, 15, 17). HPPD, Hallucinogen Persisting Perception Disorder; VSS, Visual Snow Syndrome.

The current VSS criteria did not arise from a formal consensus or validation process, but evolved from accumulating case-series experience before being incorporated into the Appendix of ICHD-3 as “Visual snow” (this is reserved for entities characterised enough to standardise research but not yet meeting the evidence threshold for its main classification). The criteria applied here (Table 5) should therefore be read as an evolving expert consensus rather than a fully validated instrument.

Step 2: Ophthalmological Assessment. OCT of the macula and peripapillary retinal nerve fibre layer (RNFL) is performed to evaluate structural retinal and optic nerve integrity. In primary VSS, OCT findings are consistently within normal limits, and the investigation serves primarily to exclude structural retinal pathology (44, 45). Electroretinography (ERG) is not routinely performed in the initial workup of all VSS patients. It is indicated selectively when retinal dystrophy, autoimmune retinopathy or photoreceptor dysfunction is clinically suspected on the basis of history or fundoscopic findings (44). Where ERG is performed, normal findings substantially reduce the probability of a primary retinal aetiology. VEPs are a neurophysiological rather than ophthalmological investigation and are typically performed in a neurophysiology setting. VEPs are not routinely required for the diagnosis of VSS but may be informative when optic pathway disease is under active consideration. In VSS, routine VEPs are often within normal limits, though impaired P1 habituation has been reported in some patients using advanced paradigms (16, 21). VEP latency delay, as seen in demyelinating disease, is not a feature of primary VSS and its presence should prompt investigation for optic neuropathy or demyelination.

Step 3: Structural Neuroimaging. Conventional brain MRI is advised in the initial evaluation of all patients with suspected VSS, primarily to exclude secondary structural causes. The minimum recommended protocol comprises T1-weighted, T2-weighted, FLAIR, and diffusion-weighted imaging (DWI) sequences, with dedicated thin-slice sequences through the posterior fossa and orbits. Gadolinium-enhanced T1-weighted imaging is indicated when inflammatory, demyelinating, or neoplastic aetiology is clinically suspected. In the majority of primary VSS patients, structural MRI is unremarkable (45, 46). MRI may additionally identify secondary causes including IIH features (empty sella, optic nerve sheath dilation, posterior scleral flattening) or cerebral venous thrombosis.

Step 4: Directed Supplementary investigations. The following investigations are performed as clinically indicated, based on the individual patient’s history, examination findings, and active differential diagnoses: (1) EEG to exclude occipital epilepsy; (2) lumbar puncture for CSF opening pressure measurement when IIH is clinically or radiologically suspected; (3) blood investigations including inflammatory markers, autoimmune screen, and toxicology as clinically appropriate; (4) audiological assessment, particularly in patients with tinnitus; and (5) structured psychiatric evaluation for anxiety, depression, and depersonalisation-derealisation disorder, given the high prevalence of psychiatric comorbidity in VSS (5) (Figure 3).

**Figure 3:**
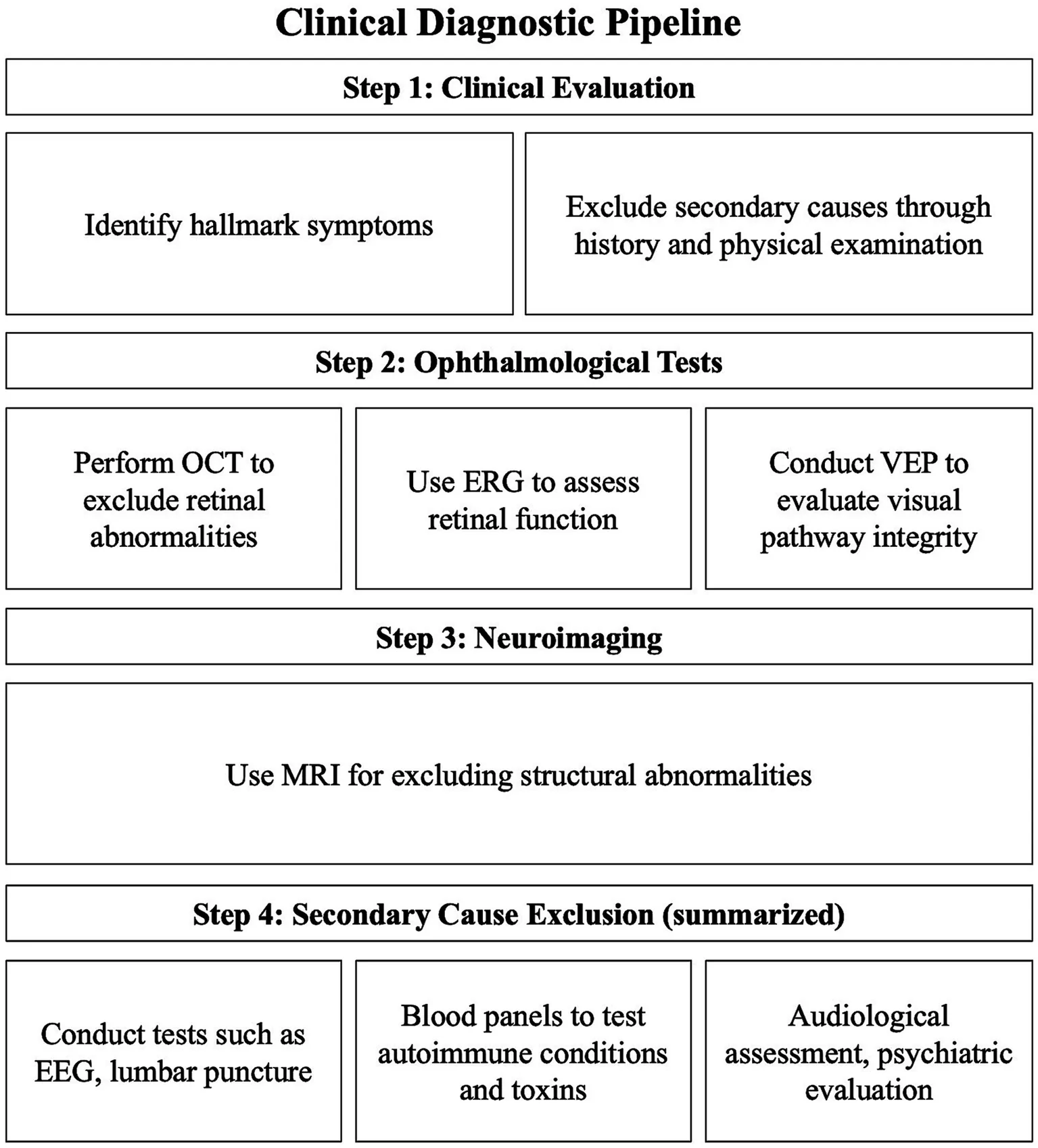
Proposed clinical diagnostic evaluation for visual snow syndrome. Stepwise approach to the evaluation of patients with suspected visual snow syndrome, including clinical assessment, ophthalmological testing, neuroimaging, and exclusion of secondary causes. VSS, Visual Snow Syndrome; OCT, Optical Coherence Tomography; ERG, Electroretinography; VEP, Visual Evoked Potentials; EEG, Electroencephalography.

#### 3.5.2 Additional research-oriented investigations

The following investigations are not part of the routine diagnostic workup for VSS and are not required to establish a clinical diagnosis. They provide mechanistic insight into the pathophysiology of primary VSS and are appropriate in dedicated research contexts or selected complex cases where additional characterisation may be clinically informative.

Resting-state and task-based fMRI has been used to characterise altered functional connectivity within visual, attentional, salience, and default mode networks in primary VSS (22, 24, 46–48). This supports a network-level conceptualisation of the syndrome. These findings have not been validated for diagnostic use and play no role in routine assessment.

18F-FDG PET findings of focal glucose hypermetabolism in the lingual gyrus and anterior cerebellum are described earlier. As these findings derive from a single-centre study, independent multicentre replication is required before any diagnostic application can be considered (20). PET currently has no role outside research protocols.

Arterial spin labelling (ASL) MRI, a non-contrast perfusion technique, has similarly identified localised increases in regional cerebral perfusion in patients with visual snow syndrome (49), broadly consistent with the hypermetabolism reported on PET. As with PET, this remains a research application with no established diagnostic role.

MEG has similarly been used to characterise cortical oscillatory dysrhythmia and thalamocortical circuit function in VSS (26, 50). As with fMRI and PET, this remains a research application only and is not transferable to routine clinical diagnosis at present. Resting-state EEG has additionally identified unstable cortical microstates in VSS, offering a lower-cost, more widely accessible complement to MEG for probing the same electrophysiological network dynamics (51). Structured diagnostics approach proposed in this article is summarised in Table 6.

**Table 6:** Structured diagnostic approach for visual snow syndrome: investigation overview.

Investigation	Setting	Diagnostic purpose	Expected finding in primary VSS
Clinical history and neurological examination	Routine	Characterise hallmark symptoms; establish ≥3-month duration; identify red flags; screen for secondary causes	VSS phenotype confirmed; no red flags; no identifiable precipitant
Optical coherence tomography (OCT)	Routine (full pathway)	Evaluate retinal and optic nerve structural integrity; exclude structural retinal pathology and optic neuropathy	Normal macular OCT and peripapillary RNFL (44, 45)
Electroretinography (ERG)	Selective (when retinal pathology clinically suspected)	Assess retinal electrophysiological function; exclude photoreceptor dystrophies and autoimmune retinopathy where clinically indicated	Normal pattern and full-field ERG findings in primary VSS (44)
Visual evoked potentials (VEP)	Selective (neurophysiology setting when optic pathway disease is considered)	Evaluate visual pathway conduction; exclude demyelination; advanced paradigms may detect impaired P1 habituation if available	Often normal; P1 habituation impairment reported in some patients (16, 21); no P100 latency delay in primary VSS
Conventional brain MRI (3 T; T1, T2, FLAIR, DWI ± gadolinium)	Routine	Exclude structural secondary causes: focal lesions, IIH features, cerebral venous thrombosis, demyelination	Normal structural MRI in primary VSS (45, 46)
EEG; lumbar puncture; blood tests; audiological and psychiatric assessment	Selective (as clinically indicated)	Exclude epilepsy; elevated ICP; autoimmune, infectious, or toxic aetiology; characterise auditory and psychiatric comorbidity	Normal where secondary cause is excluded; psychiatric comorbidity frequently identified (5)
fMRI (3 T; BOLD; resting-state and visual task paradigms)	Research / selected complex cases	Characterise altered network connectivity in visual, attentional, and default mode networks in confirmed primary VSS	Reported alterations in intra-visual and visual-attentional network connectivity (11, 25, 46)
18F-FDG PET / PET-MR	Research only	Identify proposed regional cortical glucose hypermetabolism as a pathophysiological research correlate	Lingual gyrus and anterior cerebellar hypermetabolism reported in one centre (20); independent replication required
Magnetoencephalography (MEG)	Research only	Characterise proposed cortical oscillatory dysrhythmia and thalamocortical coupling in research contexts	Cortical oscillatory dysrhythmia reported (26); not validated for routine diagnostic use

“Full pathway” applies to patients with new-onset, atypical, or evolving symptoms, red flags, or diagnostic uncertainty. Patients with a stable, long-standing presentation fulfilling the criteria in full, with no red flags, may not require OCT or brain MRI. OCT, Optical Coherence Tomography; RNFL, Retinal Nerve Fibre Layer; ERG, Electroretinography; VEP, Visual Evoked Potentials; MRI, Magnetic Resonance Imaging; FLAIR, Fluid-Attenuated Inversion Recovery; DWI, Diffusion-Weighted Imaging; IIH, Idiopathic Intracranial Hypertension; ICP, Intracranial Pressure; EEG, Electroencephalography; fMRI, Functional Magnetic Resonance Imaging; BOLD, Blood-Oxygen-Level-Dependent; PET, Positron Emission Tomography; MEG, Magnetoencephalography; VSS, Visual Snow Syndrome.

## 4 Conclusion

Visual snow syndrome is a prevalent yet substantially underdiagnosed chronic neurological condition affecting approximately 2% of the general population, with clinical impact extending beyond the visual domain. Diagnosis remains challenging due to symptom overlap with migraine aura, HPPD, and structural ophthalmological and neurological conditions. In addition, the absence of established biomarkers or consistent structural neuroimaging findings in primary cases complicates diagnosis. Even though underlying mechanisms remain incompletely understood, converging evidence from functional and electrophysiological studies is consistent with cortical hyperexcitability and network dysfunction as candidate pathophysiological mechanisms. Though this remains a hypothesis requiring further mechanistic validation rather than an established substrate.

The structured diagnostic approach presented in this review offers a clinically actionable and reproducible framework, distinguishing routine outpatient evaluation from additional research-oriented investigations. It also explicitly excludes advanced neuroimaging from standard diagnostic pathways. Broader adoption of this framework may reduce diagnostic delay, minimise unnecessary investigations, and promote a shared diagnostic language. In the absence of established pharmacological treatments and given the high prevalence of psychiatric comorbidity, a multidisciplinary, patient-centred management strategy is recommended.

Key priorities for future research include (1) elucidating the mechanisms underlying lingual gyrus hypermetabolism and network dysconnectivity, (2) identifying reliable objective biomarkers, (3) validating the structured diagnostic approach in multicentre prospective studies, (4) conducting large-scale randomised controlled trials of therapeutic interventions, including neuromodulation and targeted pharmacological agents, (5) and advancing integrated models of neurological and mental health care, alongside (6) longitudinal studies to clarify the relationship between VSS and psychiatric comorbidities.

This review has several limitations. As a narrative synthesis, it was not conducted under a pre-registered protocol, and a formal risk-of-bias or quality assessment of included studies was not performed. The evidence base is dominated by small, single-centre observational studies and case series, many without independent replication, precluding formal meta-analysis and limiting the precision of prevalence and comorbidity estimates. Finally, the VSS diagnostic criteria applied throughout this review remain an evolving expert consensus rather than a fully validated instrument, which may limit comparability across studies using differing case definitions.
