# Supplementary material for: Clinical evaluation of visual snow syndrome: a structured narrative review with proposed clinical diagnostic approach
Source: Front Neurol. 2026 Oct 12;17:1961508. doi: 10.3389/fneur.2026.1961508 (PMC13700013; doi:10.3389/fneur.2026.1961508)
Supplement: Supplementary file 1 [file Supplementary_file_1.docx]

**SUPPLEMENTARY**

**Supplementary Information 1. The search string list applied as specified.**

PubMed/MEDLINE:

*("visual snow syndrome"[Title/Abstract] OR "visual snow"[Title/Abstract]) AND ("Diagnosis"[Mesh] OR diagnos*[Title/Abstract] OR "Differential Diagnosis"[Mesh] OR "differential diagnosis"[Title/Abstract] OR symptom*[Title/Abstract] OR comorbid*[Title/Abstract] OR pathophysiolog*[Title/Abstract] OR "Neuroimaging"[Title/Abstract] OR "Magnetic Resonance Imaging"[Mesh] OR MRI[Title/Abstract] OR fMRI[Title/Abstract] OR PET[Title/Abstract] OR MEG[Title/Abstract] OR electrophysiolog*[Title/Abstract] OR "Evoked Potentials, Visual"[Mesh] OR "visual evoked potential*"[Title/Abstract] OR VEP[Title/Abstract] OR "Tomography, Optical Coherence"[Mesh] OR "optical coherence tomography"[Title/Abstract] OR OCT[Title/Abstract] OR perimetry[Title/Abstract] OR secondary[Title/Abstract]) AND english[lang]*

Scopus:

*TITLE-ABS-KEY("visual snow syndrome" OR "visual snow") AND TITLE-ABS-KEY(diagnos* OR "differential diagnosis" OR symptom* OR comorbid* OR pathophysiolog* OR neuroimaging OR "magnetic resonance imaging" OR MRI OR fMRI OR PET OR MEG OR electrophysiolog* OR "visual evoked potential*" OR VEP OR "optical coherence tomography" OR OCT OR perimetry OR secondary) AND LANGUAGE(english) AND PUBYEAR > 2019 AND PUBYEAR < 2027.* Refined by: Language = English; Timespan = 2020-01-01 to 2026-04-10.

Web of Science:

*TS=("visual snow syndrome" OR "visual snow") AND TS=(diagnos* OR "differential diagnosis" OR symptom* OR comorbid* OR pathophysiolog* OR neuroimaging OR "magnetic resonance imaging" OR MRI OR fMRI OR PET OR MEG OR electrophysiolog* OR "visual evoked potential*" OR VEP OR "optical coherence tomography" OR OCT OR perimetry OR secondary).* Refined by: Language = English; Timespan = 2020-01-01 to 2026-04-10.

**Supplementary Table 1.** **Eligibility Criteria for Study Selection**

| Criterion | Inclusion | Exclusion |
| --- | --- | --- |
| Population/condition | Human studies involving or centrally addressing visual snow syndrome | Studies not involving VSS (e.g., isolated migraine aura, HPPD without visual snow features, unrelated visual/neurological disorders) |
| Concept | Diagnosis, clinical presentation, pathophysiology, differential diagnosis, comorbidities, or diagnostic/investigative workup of VSS | Studies in which VSS is mentioned only incidentally, with no substantive diagnostic or mechanistic content |
| Publication date | 1 January 2020 – 10 April 2026 | Outside this window, except two foundational pre-2020 references (Metzler & Robertson, 2018; Luna et al., 2018) retained via manual reference tracking as seminal diagnostic-criteria papers |
| Language | English | Non-English without an available English translation |
| Publication type | Original research (clinical, observational, imaging, electrophysiological), systematic reviews, meta-analyses, and narrative/clinical reviews | Letters, editorials, notes, conference abstracts, and book chapters, unless reporting a novel secondary aetiology or diagnostically distinct finding not otherwise represented in the evidence base |
| Case reports | Included only where reporting a novel secondary cause, drug-induced aetiology, or other diagnostically distinct feature not covered by existing evidence | Single case reports duplicating findings already established by existing case series or primary studies |
| Databases searched | PubMed/MEDLINE, Scopus, Web of Science | Embase not searched directly (no institutional access); coverage substantially overlaps with Scopus/Web of Science. |
| Duplicate records | Most complete version retained where a study appears in multiple databases or as alternate-language editions | Duplicate records identified by DOI or by title where DOI was unavailable |

**Supplementary Table 2. SANRA Self-Assessment.**

| Item | Score (0–2) | Rationale |
| --- | --- | --- |
| 1. Justification of the article's importance for the readership | 2 | VSS is explicitly framed as underdiagnosed and lacking a standardised diagnostic protocol, directly motivating the review's clinical relevance |
| 2. Statement of specific goals/questions | 2 | The review explicitly aims to synthesise diagnostic and pathophysiological evidence and propose a structured clinical diagnostic approach |
| 3. Description of the literature search | 2 | Methods section details databases (PubMed/MEDLINE, Scopus, Web of Science), search strings, date restrictions, and a selection flow (Table 1) |
| 4. Referencing | 1–2 | Broad, current reference base spanning primary research and reviews through 2026 |
| 5. Scientific reasoning | 2 | Pathophysiological hypotheses are presented with explicit caveats about evidence strength (e.g., “requires further mechanistic investigation,” “remains to be established in larger cohorts”) rather than overstated conclusions |
| 6. Appropriate presentation of data | 2 | Multiple structured tables (symptom description, differential diagnosis, diagnostic criteria, investigation overview) and figures translate synthesis into clinically usable format |

**Supplementary Table 3. List of Included Primary Studies.**

| Author(s), Year | Journal | Study Type | Focus / Contribution |
| --- | --- | --- | --- |
| Adcock & Panayiotopoulos, 2012 | J Clin Neurophysiol | Review | Occipital lobe seizures (background differential diagnosis, pre-2020) |
| Aeschlimann et al., 2024 | Curr Opin Neurol | Review | Recent advances in pathophysiology/treatment |
| Aeschlimann et al., 2025 | Brain Behav | Primary research (EEG) | Unstable resting-state cortical microstates |
| Aldusary et al., 2020 | Front Hum Neurosci | Primary research (structural/connectivity MRI) | Abnormal connectivity and brain structure in VSS |
| Barral et al., 2023 | Diagnostics | Review | Differential diagnosis: aura vs. VSS |
| Braca et al., 2025 | Cephalalgia | Review | VSS vs. migraine aura debate summary |
| Brooks et al., 2024 | Invest Ophthalmol Vis Sci | Primary research | Phenotype/mechanisms of palinopsia in VSS |
| Bruninx & Lepièce, 2020 | Rev Med Liege | Review | Retinitis pigmentosa (differential diagnosis) |
| Chojdak-Łukasiewicz & Dziadkowiak, 2024 | J Clin Med | Case report + review | VSS in a migraine patient |
| Costa et al., 2022 | PLoS One | Cross-sectional (Portugal) | Prevalence, childhood onset, and emotional reactions to visual snow |
| Eren et al., 2020 | Cephalalgia | Case-control | Quantification of photophobia in VSS |
| Eren & Schankin, 2020 | Prog Brain Res | Systematic review | Pathophysiology and treatment insights |
| Fraser, 2024 | Eye | Review/commentary | Recognition and management of VSS |
| Gersztenkorn & Lee, 2015 | Surv Ophthalmol | Systematic review | Palinopsia literature review (background, pre-2020) |
| González-Ávila et al., 2024 | Neurol Sci | Cross-sectional/case series | Widening clinical spectrum of VSS with migraine |
| Göbel, 2025 | ICHD-3 (online) | Diagnostic criteria source | ICHD-3 Appendix definition |
| Hang et al., 2021 | Neuropsychiatr Dis Treat | Review + case series | Mimics of VSS |
| Harris, 2021 | Front Neurol | Case report | P1/C2 VEP potentiation and attenuation |
| Hepschke et al., 2022 | Brain Commun | Primary research (MEG) | Cortical oscillatory dysrhythmias |
| Klein & Schankin, 2021 | Front Neurol | Systematic review | VSS as a network disorder |
| Klein & Schankin, 2021 | Headache | Narrative review | VSS, perceptual disorder spectrum, migraine risk |
| Kondziella et al., 2020 | Eur J Neurol | Cross-sectional (UK) | Prevalence of VSS in the UK |
| Kondziella, 2022 | Brain Commun | Commentary/opinion | Critical perspective on VSS nosology; reassurance-seeking in lifelong VSS |
| Latini et al., 2021 | Eur J Neurol | Primary research (DTI) | White matter abnormalities (diffusion tensor imaging) |
| Luna et al., 2018 | Headache | Primary research | VEP potentiation and gamma power (foundational; manual addition, pre-2020) |
| Metzler & Robertson, 2018 | Curr Neurol Neurosci Rep | Review | Proposed VSS diagnostic criteria (foundational; manual addition, pre-2020) |
| Mulder et al., 2023 | J Vis | Primary research | Photophobia/night vision as disruptive VSS symptoms |
| Orekhova et al., 2026 | Brain Commun | Primary research (MEG) | Enhanced neural plasticity, gamma oscillations |
| Ozir et al., 2024 | Cureus | Case report | LHON with significant visual recovery |
| Patel et al., 2021 | J Neuroophthalmol | Case report/commentary | Differential diagnosis caution (“not all that flickers”) |
| Puledda et al., 2020 | Ann Clin Transl Neurol | Primary research (fMRI/MRS) | Insular and occipital changes (BOLD fMRI and MRS) |
| Puledda et al., 2020 | Neurology | Cross-sectional (n=1,100) | Large-scale clinical/phenotypic description of VSS |
| Puledda et al., 2021 | Hum Brain Mapp | Primary research (fMRI) | Disrupted connectivity in visual/attentional/salience networks |
| Puledda et al., 2021 | J Neurol Neurosurg Psychiatry | Primary research (ASL-MRI) | Localised increase in regional cerebral perfusion |
| Puledda et al., 2023 | Ann Neurol | Primary research (MRS) | Abnormal glutamatergic/serotonergic connectivity |
| Raviskanthan et al., 2022 | Front Ophthalmol | Review | Neuroimaging in visual snow |
| Romozzi et al., 2024 | Eur J Neurol | Cross-sectional (Italy) | Prevalence/characteristics in young Italian adults |
| Rusztyn et al., 2023 | J Clin Med | Review | Pathophysiology and treatment review |
| Rym et al., 2023 | Neuro-Ophthalmology | Case report/series | Ocular manifestations after methanol poisoning |
| Sampatakakis et al., 2022 | Cephalalgia | Systematic review + case series | VSS systematic review and case series |
| Schankin et al., 2014 | Brain | Cross-sectional (foundational) | Original definition distinguishing VSS from migraine aura (manual addition, pre-2020) |
| Shankar Kikkeri & Nagalli, 2026 | StatPearls | Background reference | Migraine with aura overview |
| Shemesh et al., 2026 | StatPearls | Background reference | Leber hereditary optic neuropathy |
| Shibata, 2025 | Brain Dev | Review | Brain dysfunction and therapeutic implications |
| Silva & Puledda, 2023 | Eye | Review | VSS and migraine review |
| Solly et al., 2021 | Front Neurol | Primary research (survey) | Psychiatric symptomology of VSS |
| Stern & Robertson, 2024 | Curr Pain Headache Rep | Review | Updates and narrative review |
| Strik et al., 2022 | Brain Commun | Primary research (quantitative MRI) | Ultra-high field microstructure study |
| Strik et al., 2025 | Brain Commun | Primary research (fMRI) | Altered functional connectivity strength |
| Thompson et al., 2023 | Headache | Cross-sectional (case-control) | Perceived severity and visual allodynia; longer duration linked to reduced severity |
| Traber et al., 2020 | Curr Opin Neurol | Review | Diagnosis, pathophysiology, treatment overview |
| van Dongen et al., 2021 | Eur J Neurol | Cohort/case-control | Migraine prevalence in VSS with/without HPPD |
| Van Laere et al., 2022 | EJNMMI Res | Case-control (PET/MR) | 18F-FDG PET/MR metabolic and structural changes |
| Vis et al., 2021 | Front Neurosci | Systematic review | Perception and consciousness in HPPD |
| Wall, 2010 | Neurol Clin | Review | Idiopathic intracranial hypertension (background differential diagnosis, pre-2020) |
| Wong et al., 2024 | J Neuroophthalmol | Primary research (fMRI, interventional) | VSS improvement with MBCT-modulated resting-state fMRI |
| Yoo et al., 2020 | J Clin Neurol | Case-control | Neuro-ophthalmologic findings in VSS |
| Zaroban et al., 2023 | J Neuroophthalmol | Exploratory case series | Retinal structure/electrophysiology in VSS |

**Supplementary Table 4. Notable Excluded Studies and Reasons for Exclusion.**

| Author(s), Year | Journal | Reason for Exclusion |
| --- | --- | --- |
| Barrachina-Esteve et al., 2024 (Spanish-edition DOI) | Neurología | Duplicate, same article as the English-edition version already cited |
| Rymowicz & Tsuang, 2021 | Addict Disord Their Treat | HPPD case report; redundant with existing HPPD systematic review coverage |
| Schatten et al., 2020 | Ophthalmologe | Single HPPD case report; redundant with existing HPPD coverage |
| Fenech et al., 2020 | Addict Disord Their Treat | HPPD patient survey; differential-adjacent but not VSS-specific, redundant with existing review |
| Anonymous comment, 2024 | Eye | Editorial comment on an already-cited VSS-migraine review; secondary commentary |
| Nature Reviews Neurology research highlights, 2020–2021 (x2) | Nat Rev Neurol | Secondary commentary summarising already-cited primary research |
| Mobile app for VSS, 2022 | Lecture Notes Comput Sci | Assistive technology; not diagnostic or pathophysiological in focus |
| AI text-to-image models for VSS patient perspective, 2023 | Can J Neurol Sci | Patient communication tool; not diagnostic in focus |
| Novel computational framework for VSS, 2025 | IEEE Access | Technology/AR-VR simulation tool; not diagnostic in focus |
| HPPD and non-visual perceptual disturbances, 2025 | J Psychedelic Stud | HPPD-focused on non-visual symptoms; tangential to VSS |
| Editorial: Visual Snow, Old Problem New Understanding, 2022 | Front Neurol | Editorial framing piece; limited substantive content beyond already-cited literature |
| Posterior vitreous detachment case report exploring VSS, 2025 | Headache Medicine | Niche case report; redundant with existing migraine-aura differential coverage |
| Optic nerve sheath meningioma / trabeculectomy case, 2020 | Klinika Oczna | Visual snow incidental to an unrelated primary ophthalmic diagnosis |
| Headache and Eye Pain (book chapter), 2022 | Albert & Jakobiec's Ophthalmology | General textbook chapter; VSS mentioned only in passing |
| Tinnitus, Tinnitus Disorder, and Other Phantom Perceptions (book chapter), 2024 | Textbook of Tinnitus | VSS mentioned only as an analogy to tinnitus; not substantively addressed |
| Essential readings in headache medicine: top 10 educational articles, 2025 | Headache | Generic reading list; not VSS-specific |
| Anti-glycine receptor antibody-related parkinsonism with complex visual phenomena, 2024 | Mov Disord Clin Pract | Distinct primary disease (autoimmune parkinsonism); VSS not central to the case |
